# Correlates of stunting among children in Ghana

**DOI:** 10.1186/1471-2458-14-504

**Published:** 2014-05-26

**Authors:** Eugene Kofuor Maafo Darteh, Evelyn Acquah, Akwasi Kumi-Kyereme

**Affiliations:** 1 Department of Population and Health, University of Cape Coast, Cape Coast, Ghana

**Keywords:** Stunting, Children, Ghana, Correlates

## Abstract

**Background:**

Stunting, is a linear growth retardation, which results from inadequate intake of food over a long period of time that may be worsened by chronic illness. Over a long period of time, inadequate nutrition or its effects could result in stunting. This paper examines the correlates of stunting among children in Ghana using data from the 2008 Ghana Demographic and Health Survey (GDHS).

**Methods:**

The paper uses data from the children recode file of the 2008 Demographic and Health Survey (DHS), a nationally representative cross sectional survey conducted in Ghana. A total of 2379 children under five years who had valid anthropometric data were used for the study. Data on the stunting of children were collected by measuring the height of all children under six years of age. A measuring board produced by Shorr Productions was used to obtain the height of the children. Children under 2 years of age were measured lying down on the board while those above 2 years were measured standing. In the DHS data, a z-score is given for the child’s height relative to the age. Both bi-variate and multi-variate statistics are used to examine the correlates of stunting.

**Results:**

Stunting was common among males than females. Age of child was a significant determinant of stunting with the highest odd of stunting been among children aged 36–47 months. Region was significantly related to stunting. Children from the Eastern Region were more likely to be stunted than children from the Western Region which is the reference group (OR = 1.7 at p < 0.05). Number of children in household was significantly related to stunting. Children in households with 5–8 children were 1.3 times more likely to be stunted compared to those with 1–4 children (p < .05). Mother’s age was a significant predictor of stunting with children whose mothers were aged 35–44 years being more likely to be stunted.

**Conclusion:**

Culturally appropriate interventions and policies should be put in place to minimise the effects of the distal, proximal and intermediate factors on stunting among under 5 children in Ghana.

## Background

Malnutrition is a primary factor in over 50% of the annual deaths of children under 5 years of age who die from preventable causes [1]. It is directly or indirectly responsible for 54% of the 10.8 million deaths per year and contributes to every second death associated with infectious diseases among children under five years of age in developing countries [2].

In sub-Saharan Africa, prevalence of malnutrition is among the highest in the world with about 42% of children under five being stunted [3]. Infants and pre-school children are most vulnerable to retardation in growth as a result of malnutrition [4]. Adequate nutrition is necessary for appropriate growth and physical development from conception to adulthood [5]. This is essential to enhance optimal working competence, standard reproductive performance and sufficiency of immune mechanism which present resistance to infections [5]. The deficiency in the adequate nutrition required at each stage of human life for optimum growth and development may lead to malnutrition.

Stunting results in a linear growth retardation and results from inadequate intake of food over a long period of time that may be worsened by chronic illness [6]. Children are considered too short for their age when they have height-for-age below minus two standard deviations (-2SD) from the median of the reference population and those whose height-for-age are below minus three standard deviations (-3SD) from the reference population median are considered severely stunted [7]. Inadequate nutrition over a long period of time or the effects of recurrent or chronic illness could result in stunting [7].

The International community has made several efforts to reduce the malnutrition burden especially in developing countries. The Millennium Development Goals (MDGs) one and four which intend to eradicate extreme poverty and hunger and reduce child mortality respectively by two-thirds by the year 2015 were set to aid in the reduction of the malnutrition burden [8]. Also, there have been various interventions such as exclusive breastfeeding and micronutrient supplementation to reduce child malnutrition in developing countries [9].

In Ghana, there have been approaches such as the capitation grant, school feeding programme and the Livelihoods Empowerment against Poverty Programme (LEAP) put in place to help reduce the levels of food insecurity, malnutrition and poverty in targeted communities and ensure maximum benefits to children due to their vulnerability [8]. The contributions of these approaches to the reduction of child malnutrition are however yet to be confirmed.

Twenty-eight per cent of children under five in Ghana are suffering from stunted growth [7]. Though there has been a reduction from the 2003 figure of 29.9% [7], the current rate is still high. Despite the high level of stunting, many nutrition actions have not addressed child malnutrition in a broader way. A number of studies have identified factors which put children at risk of malnutrition [7,10,11].

A few studies have been conducted on stunting using both community level and national data in Ghana [12,13]. However, there is a paucity of literature on the correlates of stunting based on nationally representative data set in the country. This paper, therefore, examines the correlates of stunting among children in Ghana using data from the 2008 Demographic and Health Survey.

### Conceptual framework

**Figure 1:**
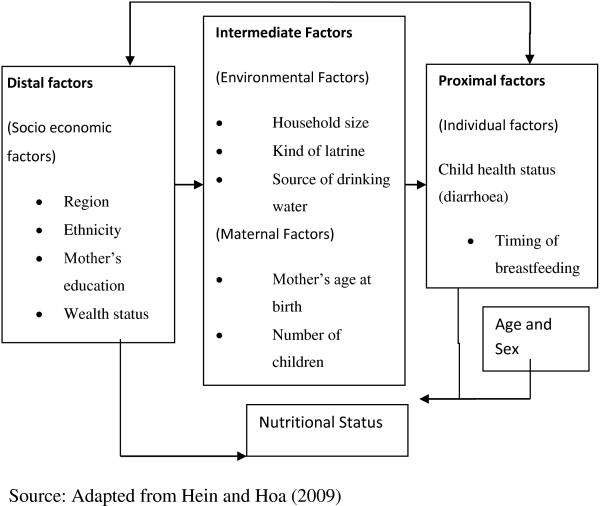
Conceptual framework.

There are several models to explain the determinants of child health. For the purpose of this paper, however, the conceptual hierarchical framework for analysing determinants of nutritional status developed by Hien and Hoa [14] was adapted (see Figure 1). It was developed to explain child malnutrition in areas with high prevalence of child malnutrition especially in developing countries. The proponents postulate that once a child lives in a society, changes in the society will affect the child’s nutritional status directly or indirectly through his or her socio-demographic, environmental, maternal and individual factors.

This framework provides a way of understanding how the different factors affecting child malnutrition are linked. It shows how the distal factors act through the intermediate and proximal factors to affect a child’s nutritional status. It brings out larger concepts that interrelate to bring an outcome. Under each larger concept are specific variables that could be measured to achieve the purpose of the study. The variables used for the study were region, child’s age, household size, source of drinking water, kind of latrine, ethnicity, mother’s level of education, age, number of children, wealth status, child’s health status and timing of breastfeeding.

## Methods

The paper uses data from the children recode file of the 2008 Demographic and Health Survey, a nationally representative cross sectional survey conducted in Ghana. The data for this study were requested online from the Demographic and Health Survey website. An approval was given to download the data. The Ghana Health Service Ethical Review Committee in Accra gave ethical approval for the study protocol. The STATA data file of the children recode file was downloaded for the study.

The survey was conducted with a sample of more than 12,000 households chosen nationally. A two-stage sample design was used with the first stage involving choosing sample points or clusters from a restructured master sampling frame created from the 2000 Ghana Population and Housing Census. In all 412 clusters were chosen from the master sampling frame.

The second stage of the assortment involved the systematic sampling of 30 of the households scheduled in each cluster. Finally a sample of 12,323 households was selected for the survey. With the Household Questionnaire, each household selected for the GDHS was eligible for interview. Height and weight measurements of children under the age of five years were taken in the households selected for the individual interview.

The number of occupied households successfully interviewed was 11,778, yielding a household response rate of 99 percent. In the households selected for individual interview in the survey, a total of 5,096 eligible women were identified; interviews were completed with 4,916 of these women, yielding a response rate of 97 percent. In the same households, a total of 4,769 eligible men were identified and interviews were completed with 4,568 of these men, yielding a response rate of 96 percent [7]. There were 2,912 children under age five in the GDHS households of which 87% had their applicable height and weight measurement obtained [7]. Data on the nutritional status of children were collected by measuring the height and weight of all children under six years of age. A lightweight, electronic seca scales with a digital screen, designed and manufactured under the guidance of the United Nations Children’s Fund (UNICEF) was used to collect the data on weight, while a measuring board produced by Shorr Productions was also used to obtain the height of the children [7].

Children under 2 years of age were measured lying down on the board while those above 2 years were measured standing. Collected data were entered using CSPro, a programme specially developed for use in DHS surveys [7]. All data were entered twice for 100 percent verification. A total of 2379 children under five years who had valid anthropometric data were used for the study.

In the DHS data, a z-score is given for the child’s height relative to the age. It is defined as the number of standard deviation units from the median height among children at that age in an international reference population. Stunting is defined as a z-score lower than -2. The anthropometric data were made up of z-scores of the children’s height-for-age, weight-for-height and weight-for-age. The z-score was divide by 100 to get a single digit. For stunting, children with z-score of -201 to -599 for height-for-age were considered stunted and those with z-score of -199 to -592 were considered to be stunted.

The dependent variable for this study was stunting. Stunting was coded into 1 stunted and 0 not stunted. Region, place of residence, child’s age, household size, source of drinking water, kind of latrine, ethnicity, and mother’s level of education, wealth and marital status were the independent variables. STATA version 11 was used to recode the dependent and independent variables. Age in months was group into less than 6 months, 6–11, 12–23, 24–35, 36–47 and 48–59. Educational level was put into four categories; no education, primary level, middle or Junior Secondary School level and Senior Secondary School or higher education. Marital status was divided into three categories: never married, married or living together with a man and divorced or widowed. Region of residence was made up of the ten regions of Ghana including Ashanti, Western, Eastern, Volta, Northern, Brong Ahafo, Central, Greater Accra, Upper East and Upper West Regions. The place of residence was divided into urban and rural. Ethnicity was recoded into five ethnic groups namely Akan, Ga/Dangme, Ewe, Mole-Dagbani, and Others. Child’s age was recoded into less than six months, 6–23 months and 24–59 months based on the literature. Sex of child was divided into male and female. Wealth status was divided into five categories namely poorest, poorer, middle, richer and richest. The household size of the children was recoded into 1–4, 5–8, 9 and above based on the literature. The source of drinking water was recoded into four categories including pipe, well, spring or river or rainwater and tanker or bottle or sachet water. Type of toilet facility was also recoded into water closet, pit latrine and bush or no facility. The dataset was reorganised by dropping and keeping some of the variables to suit the objectives of the study. Descriptive statistics was used to describe the variables of the study. Binary logistic regression was used to examine the determinants of stunting. Binary logistic regression was used because the dependent variable is dichotomous. Three stepwise models were constructed for the study, based on the categorisation of the independent variables into distal, intermediate and proximal factors. Model one looked at the child’s age, sex, region, ethnicity, mother’s level of education and wealth status. The second model was fitted to assess how the variables in the first model would react when the intermediate variables including household size, number of children and mother’s age are added. Model three was fitted to examine the effects of proximal factors such as incidence of diarrhoea on stunting among children. This technique was informed by the conceptual framework for the study. To ensure that variables were not co-linear, we run correlation analysis for variables for which we suspected some correlation such as region and ethnicity. The results showed weak and non-significant correlation (data not shown).

## Results

### Background characteristics of children

The background characteristics of children whose anthropometric data were taken during the survey are presented in Table 1. The proportion of stunting among the children was 27.5 per cent. Twenty one percent of the children were aged 12–23 months while 20 percent were aged 48–59 months. More than 50 percent of the children were females and the rest were males. Twenty percent of the children were from Ashanti Region while 3 percent were from the Upper West. The most dominant ethnic group of the children was Akan (48%) with 21 percent of them being Mole-Dagbani.

**Table 1:** Background characteristics of children

**Variable**	**Category**	**Percent**
Proportion of stunted children	NA	27.5
Age (N = 2325)	Less than 6 months	8.8
	6-11months	11.4
	12-23 months	21.4
	24-35 months	19.3
	36-47 months	18.8
	48-59 months	20.5
Sex of child (N = 2379)	Females	50.8
	Males	49.2
Region (N = 2325)	Western	9.3
	Central	9.1
	Greater Accra	11.5
	Volta	9.1
	Eastern	8.2
	Ashanti	19.7
	Brong Ahafo	10.5
	Northern	15.2
	Upper East	4.8
	Upper West	2.6
Ethnicity (N = 2230)	Akan	47.9
	Ga/Dangme	5.0
	Ewe	13.3
	Mole-dagbani	21.4
	Others	12.4
Mother’s Educational level	No education	32.9
(N = 2323)	Primary	24.3
	Middle/JHS	34.9
	Secondary and above	7.9
Wealth Status (N = 2325)	Poorest	25.6
	Poorer	22.9
	Middle	17.8
	Richer	19.5
	Richest	14.2
Household size (N = 2325)	1-4	35.2
	5-8	53.1
	9 and above	11.7
Source of drinking water (N = 2311)	Pipe	35.6
	Well	43.6
	Spring/river/rainwater	14.5
	Tanker/bottle/sachet	6.3
Type of toilet facility (N = 2309)	Water closet	8.6
	Pit latrine	63.6
	Bush/no facility	27.8
Number of children (N = 2329)	1-4	71.6
	5-8	25.0
	9-14	3.4
Mother’s age (N = 2345)	15-24	22.1
	25-34	49.0
	35-44	25.6
	45 and above	3.3
Timing of breastfeeding (N = 2323)	<1 hour	53.4
	1 hour	11.7
	2^nd^ - 23^rd^ hour	18.2
	< 6 days	16.0
	6^th^ - 14^th^ day	0.7
Incidence of diarrhoea (N = 2332)	Yes	20.9
	No	79.1

Source: Computed from 2008 GDHS dataset.

Thirty-five percent of the children had mothers with up to middle school education while 8 percent had mothers with secondary or higher education. About one in four of the children were from the poorest households, whereas to 14 percent of children were from the richest households. Fifty-three percent of the children were from households with 5–8 members while 35 percent were from households with 1–4 members. The most common source of drinking water for the children was well water (44%) with 36 percent having access to piped water. Sixty-four percent of the children were from households that used pit latrine as their type of toilet facility while nine percent were from households that used water closet as their toilet facility.

Seventy-two percent of the children had mothers who had given birth to 1–4 children while 3 percent had mothers who had given birth to 9–14 children. Forty nine per cent of the children had mothers aged 25–34 years while 3 percent had mothers aged 45 and above. More than half of the children were breastfed less than an hour after birth compared to less than one percent of those who were breastfed 6–14 days after birth. Twenty one percent of the children had had diarrhoea two weeks before the survey.

### Multivariate analysis

Logistic regression was used to further analyse the determinants of stunting with three stepwise models. In Model 1 distal factors were used to estimate their effect on stunting. Model 2 shows the relationship between distal and intermediate factors and stunting and model three is used to estimate the combined effects of distal, intermediate and proximal factors on stunting. The logistic regression results of the determinants of stunting are presented in Table 2.

**Table 2:** Determinants of stunting

**Variable**	**Model 1**	**Model 2**	**Model 3**
*Distal factors*			
*Age in months*			
Less than 6 (ref)	1.00	1.00	1.00
6-11	2.73**	2.74**	2.69**
12-23	7.84**	7.95***	7.84***
24-35	9.89***	10.24***	10.11***
36-47	10.42***	11.14***	11.10***
48-59	9.27***	10.17***	10.18***
*Sex*			
Male	1.00	1.00	1.00
Female	0.85	0.84	0.84
*Educational level*			
No education (ref)	1.00	1.00	1.00
Primary	1.31	1.27	1.28
Middle/JHS	1.13	1.23	1.14
Secondary and above	1.14	1.14	1.16
*Wealth Status*			
Poorest (ref)	1.00	1.00	1.00
Poorer	0.89	0.89	0.87
Middle	0.60**	0.60**	0.59**
Richer	0.43***	0.43***	0.43***
Richest	0.29***	0.32***	0.32***
*Ethnicity*			
Akan (ref)	1.00	1.00	1.00
Ga/Dangme	0.70	0.70	0.70
Ewe	0.57	0.55**	0.55**
Mole-dagbani	0.84	0.83	0.82
Others	0.99	1.02	1.03
*Region*			
Western (ref)	1.00	1.00	1.00
Central	1.56	1.48	1.48
Greater Accra	0.93	0.88	0.88
Volta	1.18	1.19	1.20
Eastern	1.67**	1.66**	1.65
Ashanti	0.99	0.97	0.97
Brong Ahafo	0.77	0.76	0.75
Northern	1.22	1.16	1.14
Upper East	1.30	1.28	1.29
Upper West	0.72	0.69	0.68
*Intermediate factors*	NA		
*Household size*	NA		
1-4 (ref)	NA	1.00	1.00
5-8	NA	0.79	0.79
9+	NA	0.98	0.99
*Mother’s age*			
15-24 (ref)	NA	1.00	1.00
25-34	NA	0.72**	0.72**
35-44	NA	0.56**	0.56***
45 an above	NA	0.54	0.54
*Number of children*			
1-4 (ref)	NA	1.00	1.00
5-8	NA	1.33	1.34**
9-14	NA	1.65	1.66
*Proximal factor*			
*Incidence of diarrhoea*			
Yes	NA	NA	1.00
No	NA	NA	1.10

Source: Computed from 2008 GDHS dataset: ***p < 0.001, **p < 0.05.

P-values in parenthesis; ref = reference.

There was a significant relationship between age of child and stunting. For instance, in model 1, children aged 36–47 months were 10.4 times more likely to be stunted compared to those aged less than six months (.001). The effects of age increased to 11.4 times compared to the reference (.001) with the addition of intermediate factors in model 2 (see Table 2). There was a significant relationship between household wealth and stunting among children. For instance, children belonging to richer households were 0.43 times less likely to experience stunting compared to the poorest (p < .001). This effect was consistent across all the three models (see Table 2). With the addition of intermediate and proximal factors in models 2 and 3 respectively, the effect between ethnicity and stunting became significant with Ewes being less likely to be stunted compared to Akans (OR = 0.55, p < .05). There was a significant relationship between region and stunting with children from the Eastern Region more likely to be stunted compared to those from the Western Region (OR = 1.7, p < 0.05) in model 1. The same effect was observed after the addition of the intermediate factors in model 2 (see Table 2). Mother’s age was significantly related to stunting among children. The results show that children whose mother’s were aged 25–34 years were less likely to be stunted compared to those whose mothers were aged 15–24 years. The effect of mother’s age on stunting remained the same after the addition of the proximal factor in model 3. A significant relationship was observed between number of children and stunting among children. For instance, it was observed that children whose mothers had 5–8 children were more likely to be stunted compared to those whose mothers had 1–4 children (OR = 1.3, p < 0.05).

## Discussion

The study sought to examine the correlates of stunting among under 5 children in Ghana using the conceptual hierarchical framework for analysing determinants of nutritional status developed by Hien & Hoa [14]. The findings of the study suggest that some distal, proximal, and intermediate factors are significantly associated with stunting among under 5 children in Ghana. We discuss the correlates of stunting among under 5 children according to the concepts used in the framework.

### Distal factors

*Ethnicity* plays a significant role in stunting among children under 5. The study observed some variations in stunting among ethnic groups, with children from the Ewe ethnic group been less likely to be stunted compared to Akans. Our study corrobrates the findings of Badasu [15] and Gyimah [16] who have reported that there are variations in stunting among ethnic groups in Ghana. These variations could be attributed to the fact that some ethnic groups in Ghana have beliefs which prevent pregnant women and infants from eating some foods. These foods may contain some nutritional elements needed for the optimal growth and development of the child.

*Region of residence* was significantly related to stunting. For instance, it was observed that children from the Eastern Region were more likely to be stunted compared to those from the Western Region in model 1. The differences between region of residence and stunting could be due to the differences in values, beliefs, culture and conditions that exist within each region or ethnic group [13].

*Household wealth status* was significantly correlated with stunting among under 5 children in Ghana. We observed a significant relationship between household wealth and stunting, with children from richer households being less likely to suffer from stunting. This finding corroborates observation made in previous studies regarding wealth status of child’s household and stunting [17-19]. This relationship could be explained by the fact that rich people are able to afford good living conditions that may improve the child’s health including nutrition [20].

### Intermediate maternal factors

*Mother’s age* was found to play a significant role in predicting stunting among children in Ghana. The results of the study is consistent with a previous study which revealed that mothers who give birth at very early age are likely to have children with low weight at birth [13]. For instance, children whose mothers were aged 25–34 years were less likely to be stunted compared to those aged 15–24 years. This could be as a result of the fact that young mothers require adequate nutrition to fully grow into adults; thus, they struggle with their children over the little food the mother eats [14].

*Number of children in household.* We observed that children from households with 5–8 children were more likely to be stunted compared to those with 1–4 children. This could be due to the large level of consumption of resources in the household [10,21]. The findings of our study corroborates previous studies which have observed that children with many siblings are more likely to suffer from malnutrition [14,22].

### Proximal

*Age of child* was found to be a significant determinant of stunting with children aged 24 months and above being more likely to be stunted. This finding is consistent with previous studies which show that stunting is high among children of that age and this situation has been attributed to the fact that these children have already been introduced to complementary feeding [7].

This study is limited by its cross-sectional nature and hence causal inferences cannot be made. However, the study has some compelling strengths. These include the large sample size and the representativeness of the sample which gave sufficient power and enhances its generalisability to other settings.

## Conclusion

The proportion of stunting among children in Ghana is relatively low (27%) compared to that of the sub-region (42%). Stunting among children is influenced by distal, proximal and intermediate factors such as age, ethnicity, mother’s age, number of children in household, wealth status, and region. To improve the nutritional status of children in Ghana, factors that are significantly correlated with stunting should be addressed. Firstly, the effects of the distal factors like ethnicity and region of residence could be reduced by putting in place culturally appropriate interventions and policies to promote the consumption of nutritious foods which are not consumed by nursing mothers and their children. These interventions and policies should aim at changing peoples attitudes towards some of societal values, beliefs and culture. Secondly, pro-poor policies should be implemented to reduce the effect of household wealth on stunting. Thirdly, programmes and policies aimed at delaying age at child birth and promoting small family sizes should be strengthened to minimise the effects of intermediate factors on stunting among under 5 children in Ghana. Finally, programmes aimed at promoting proper child feeding practices should be strengthened to reduce the effect of complimentary feeding on stunting among children under 5.

## Competing interests

The authors declare that they have no competing interests.

## Authors’ contributions

EKMD conceived the study, conducted data analysis and interpretation as well as drafted the first version of the manuscript. EA conducted some aspects of the data analysis and interpretation. EKMD, EA and AKK revised the manuscript for important intellectual content and gave consent for the version to be published. All authors have read and approved the final manuscript.

## Pre-publication history

The pre-publication history for this paper can be accessed here:

http://www.biomedcentral.com/1471-2458/14/504/prepub
